# Identification of additional body weight QTLs in the Berlin Fat Mouse BFMI861 lines using time series data

**DOI:** 10.1038/s41598-024-56097-z

**Published:** 2024-03-14

**Authors:** Manuel Delpero, Paula Korkuć, Danny Arends, Gudrun A. Brockmann, Deike Hesse

**Affiliations:** 1 Albrecht Daniel Thaer-Institut für Agrar- und Gartenbauwissenschaften, Humboldt-Universität zu Berlin, Berlin, Germany; 2 Department of Applied Sciences, Northumbria University, Newcastle Upon Tyne, UK

**Keywords:** Genetics, Heritable quantitative trait, Quantitative trait loci, Physiology, Metabolism, Diseases, Endocrine system and metabolic diseases, Obesity

## Abstract

The Berlin Fat Mouse Inbred line (BFMI) is a model for obesity and metabolic syndrome. The sublines BFMI861-S1 and BFMI861-S2 differ in weight despite high genetic similarity and a shared obesity-related locus. This study focused on identifying additional body weight quantitative trait loci (QTLs) by analyzing weekly weight measurements in a male population of the advanced intercross line BFMI861-S1 x BFMI861-S2. QTL analysis, utilizing 200 selectively genotyped mice (GigaMUGA) and 197 males genotyped for top SNPs, revealed a genome-wide significant QTL on Chr 15 (68.46 to 81.40 Mb) for body weight between weeks 9 to 20. Notably, this QTL disappeared (weeks 21 to 23) and reappeared (weeks 24 and 25) coinciding with a diet change. Additionally, a significant body weight QTL on Chr 16 (3.89 to 22.79 Mb) was identified from weeks 6 to 25. Candidate genes, including *Gpt*, *Cbx6*, *Apol6*, *Apol8*, *Sun2* (Chr 15) and *Trap1*, *Rrn3*, *Mapk1* (Chr 16), were prioritized. This study unveiled two additional body weight QTLs, one of which is novel and responsive to diet changes. These findings illuminate genomic regions influencing weight in BFMI and emphasize the utility of time series data in uncovering novel genetic factors.

## Introduction

More than one billion of the world’s population is obese, and the incidence is further increasing^1,2^. Modern lifestyle characterized by overconsumption of energy dense food and reduced physical activity contributes to this epidemic. However, the stage is set by genetic variation which influences the development and progress of the disease.

Animal models and, in particular, mouse models are essential to reveal the genetic contributions of complex diseases and further investigate the function of these genetic discoveries^3^. Body weight of animals is often collected regularly and routinely during animal experiments to monitor animal health. Usually in quantitative trait locus (QTL) studies, time series data like weekly body weight are not considered and only data such as final body weight as endpoint measurement are analyzed. However, QTL mapping with body weight measurements collected weekly could be of particular interest to examine body weight gain in obesity research to identify additional genes linked to obesity and related metabolic disorders that exhibit their effect more prominently during development or disease progression and could fade or be lost at later timepoints^4^.

The Berlin Fat Mouse population was selected for obesity. After 58 generations of selection, different Berlin Fat Mouse Inbred (BFMI) lines were generated through repeated brother-sister mating^5^. In a cross between the most obese inbred line BFMI860 (BFMI860/Hber, MGI:5448848) and the lean control strain C57BL/6NCrl (B6N), we have previously identified a recessive genetic defect at a locus on chromosome (Chr) 3 accounting for 40% of the variance in adipose tissue weight at 6 weeks^6^. This juvenile obesity locus (*jObes1*) is fixed in all BFMI sublines. Recent studies identified the gene *Bbs7* as the causal gene in this locus^7,8^ whose defect results in the Bardet-Biedl syndrome in humans, a ciliopathy associated with obesity^9^.

To detect novel genetic factors contributing to body weight, we used the inbred lines BFMI861-S1 (S1) and BFMI861-S2 (S2) which were created from their predecessor BFMI861 (BFMI861/Hber, MGI:5448851). These two lines were derived from one parental line that was divided into two sublines only after four generations of inbreeding. Consequently, these two sublines share a considerable genetic resemblance, and any observed variations in phenotype can be attributed to the limited remaining genetic diversity. Although genetically close, the S1 and S2 lines are quite different with respect to metabolic traits^5^. In particular, the S1 line shows higher body weight, hepatic fat storage, low insulin sensitivity, and impaired glucose tolerance. In contrast, S2 is insulin sensitive despite being obese^5^.

In a previous study, we used an advanced intercross population (AIL) between the BFMI861-S1 line and the reference strain B6N to discover further regions involved in body weight regulation. By applying a variation of multiple QTL mapping approaches (MQM) which adjust for the large effect of *jObes1* (by including it as cofactor into the model), we were able to identify a hidden body weight QTL on Chr 6^10^.

In another study, an AIL population was used, which was generated from an initial cross between the BFMI861 lines S1 and S2 (AIL BFMI861-S1 x BFMI861-S2), to identify more genetic loci accounting for the observed phenotypic difference in traits of the metabolic syndrome of the S1 line^11^. The advantage of crossing the two BFMI lines is that it naturally corrects for the significant effect of the *jObes1* locus on body weight, as both lines carry the high allele. Furthermore, this population may reveal hidden minor QTLs that contribute to weight variability and have not been discovered previously. As a result, three novel QTLs for traits of the metabolic syndrome (Chr 3: gonadal adipose tissue weight, blood glucose, Chr 15: gonadal adipose tissue weight, Chr 17: gonadal adipose tissue weight, liver weight, blood glucose concentration, liver triglycerides) and one QTL for body weight on Chr 16 were successfully identified using end point measurements at week 25^11^.

In the current study, we focused not only on the endpoint measurement but on time series body weight data that were collected in this AIL population weekly from week 3 until week 25. This data allowed us to identify additional body weight QTLs that contribute to the overall obese phenotype peculiar to the BFMI lines in addition to the known major QTL *jObes1* on Chr 3 and the other QTLs on Chr 6 and 16.

## Material and methods

### Mouse population

We used male mice from the 10th generation of the AIL population BFMI861-S1 x BFMI861-S2, which originated from an initial breeding between a S1 male and a S2 female, followed by successive rounds of random mating in each subsequent generation^5^. The randomization of mating pairs was done using the RandoMate program^12^.

### Animal husbandry and phenotyping

The German Animal Welfare Authorities granted approval for all experimental treatments involving mice under the reference number G0235/17 and reported in accordance with ARRIVE guidelines. All methods were performed in accordance with the relevant guidelines and regulations. The mice were maintained in standard conditions, with a 12-h light–dark cycle (lights turned on at 0600 h), and at a controlled temperature of 22 ± 2 °C. Furthermore, the mice were provided with ad libitum access to both food and water. Mice received a standard diet until week 20 (16.7 MJ/kg of metabolizable energy, 11% from fat, 26% from protein, and 53% from carbohydrates, V1534-000, ssniff EF R/M; Ssniff Spezialdiäten GmbH, Soest, Germany), followed by two weeks of a high-fat, low-carbohydrate diet (16.9 MJ/kg of metabolizable energy, 34% from fat, 19% from protein, and 47% from carbohydrates, C1057; Altromin Spezialfutter GmbH & Co. KG, Lage, Germany) to increase obesity but to protect β-cells and finally three additional weeks of high-fat, high-carbohydrate diet feeding until week 25 (21.9 MJ/kg of metabolizable energy, 28% from fat, 20% from protein, and 40% from carbohydrates^13^ to enhance metabolic differences^11^.

Body mass was recorded weekly using a standard laboratory scale between the age of 3 (after weaning) and 25 weeks (Supplementary File 1). Outliers were defined as individuals which have a measurement that deviates from the population mean by more than three standard deviations and were removed from the data.

### Genotyping

Among the 397 male mice subjected to phenotyping, 200 mice, representing the extreme ends of the phenotypic distributions for gonadal adipose tissue weight and liver weight, were chosen for genotyping using the GigaMUGA Array^11^. Due to high genetic similarity of the parental lines S1 and S2, only 5,171 (distribution: Supplemental Fig. 1B in^14^) out of 143,259 SNPs on the array were informative and passed the quality control^11^ (Supplementary File 2). Genomic positions are given according to the Mouse Genome Version MM10, GRCm38.p6.

To reduce the potential bias in estimating allele effect sizes caused by selective genotyping, the remaining 197 males of the AIL population were genotyped for two top markers identified in an initial QTL scan as being associated with body weight (see QTL mapping section). For these markers, KASP genotyping assays were developed (Supplementary Table 1).

### QTL mapping and candidate gene prioritization

QTL mapping was conducted for each body weight time point in a two-step process. Initially, a QTL scan was carried out using the 200 AIL males genotyped with the GigaMUGA array. Subsequently, a final QTL scan was conducted, incorporating all male animals (genotyped with both GigaMUGA and KASP methods).

Multiple testing correction was performed using Bonferroni method^15^ and the number of independent SNPs as determined by simpleM^16^ which was estimated as m_Eff_ of 849 using a window size of 820. P-values were converted to LOD scores using LOD = −log10(p-value). LOD scores exceeding 4.9 and 4.2 were considered as highly significant (p < 0.01) and significant (p < 0.05), respectively. To establish the 95% confidence interval for a QTL, a 1.5 LOD drop from the top SNP position was applied^17^. For each week of body weight measurement, the start and end positions of this interval were defined as the positions of the first SNP upstream or downstream of the 1.5 LOD-drop confidence interval. The final QTL interval was defined by taking the smallest start and highest end point across all measured weeks. At this, the 1.5 LOD drop was calculated considering only the markers from the 200 males genotyped with the GigaMUGA array.

Mouse genes were obtained from Ensembl release 102 counting 21,357 protein-coding genes. Positional candidate genes in each QTL region were investigated for polymorphic DNA sequence variants between the parental lines S1 and S2 mice also considering 1000 bp up- and downstream of the genes. Afterwards, candidate genes were prioritized as described in^11^. This was done using expression data from microarrays, pathway information using the KEGG database information, and consequences of sequence variants on gene transcripts (missense mutations including SIFT score information, mutations in splice site, UTRs, promotor, CTCF binding sites and enhancers), as estimated using the Ensembl Variant Effect Predictor^18^. Gene expression of liver and gonadal adipose tissue was measured using Clariom S assay in parental lines S1 and S2^11^. Fold changes (FC) were calculated and p-values were adjusted using Benjamini–Hochberg correction. Differently expressed genes were defined as genes with an adjusted p-value < 0.05.

### Detection of ChoRE motifs

Due to the diet-sensitive nature of the QTL on Chr 15, positional candidate genes were further scanned on the presence of carbohydrate-response elements (ChoRE). Based on previous research, it has been shown that expression of genes with a ChoRE motif can be induced upon glucose, adenosine-containing molecules, and other physiological cues^19^. To investigate the presence of ChoRE motifs, all genes present in the chromosome 15 QTL were determined using biomaRt. The R package GenomicFeatures and associated R data packages containing the MM10 mouse genome sequence and annotation (TxDB.Mmusculus.UCSC.mm10.endGene) were used to extract 2000 bp upstream of the genes transcription start site. Two ChoRE motifs weight-matrices were defined based on the identified ChoRE-a CACGAG(N)_5_CACGAG and ChoRE-b CACACC(N)_5_CACGCG motifs determined by Yu and Luo^19^. Using R function matchPWM (min.score = 90%, R package Biostrings), the 2000 bp upstream of each gene were scanned to identify presence of the ChoRE-a and ChoRE-b motifs.

## Results

### QTL mapping

QTL mapping was performed for body weight of the AIL population with data collected once a week from week 3 until the end of the experiment at week 25. QTL analysis on selectively genotyped 200 AIL males revealed significant loci on Chr 15 and 16. The follow-up QTL analysis after KASP genotyping including all 397 males, confirmed the two QTLs and provided true estimates for the genetic effect size (Table 1).

**Table 1:** Mean body weight in g and LOD of body weight QTLs for the top SNP identified in the AIL (BFMI861-S1 × BFMI861-S2) in up to 397 mice between week 3 and 25.

QTL Chr15				Week	QTL Chr16			
Region: 68.461.862—81.398.815 bp					Region: 3.892.297—22.791.365 bp			
top SNP: UNC25922623 (77.362.610 bp)					top SNP: UNCHS041907 (16.995.303 bp)			
S1/S1	H	S2/S2	LOD		S1/S1	H	S2/S2	LOD
11.02	10.63	10.02	2.47	3	11.03	10.44	10.18	2.23
17.35	16.95	16.31	1.54	4	17.32	16.75	16.02	2.96
23.36	22.82	22.13	1.91	5	23.33	22.64	21.95	3.35
26.91	25.97	25.20	3.34	6	26.77	25.83	25.02	**5.52**
29.50	28.40	27.71	3.26	7	29.44	28.34	27.41	**7.16**
31.97	30.76	29.91	4.15	8	31.86	30.68	29.42	**9.97**
34.01	32.70	31.43	**5.96**	9	33.59	32.46	31.30	**8.08**
35.66	34.06	32.75	**5.96**	10	35.22	33.93	32.52	**9.52**
37.25	35.16	33.79	**6.64**	11	36.50	35.02	33.50	**9.91**
38.83	36.69	35.13	6.77*	12	38.14	36.44	34.74	**10.82**
40.34	37.78	36.27	**6.60**	13	39.42	37.64	35.68	**10.85**
41.53	38.90	37.19	**6.93**	14	40.75	38.65	36.67	**11.59**
42.23	39.72	37.92	**6.60**	15	41.61	39.46	37.56	**10.91**
43.40	40.82	38.79	**7.08**	16	42.70	40.47	38.49	**10.93**
44.14	41.60	39.41	**6.77**	17	43.58	41.27	39.28	**10.50**
40.93	38.40	36.34	**7.41**	18	40.45	38.15	36.19	**11.84**
44.47	41.00	39.09	**6.80**	19	43.27	41.12	38.77	**9.97**
44.41	41.17	39.01	**7.81**	20	43.41	41.16	38.80	**11.49**
43.73	41.45	39.72	3.83	21	43.20	41.20	39.49	**6.13**
45.16	42.95	40.97	3.93	22	44.83	42.58	40.97	**5.69**
47.22	45.06	43.60	3.56	23	47.35	44.99	43.53	**6.32**
48.61	46.78	45.25	**4.81**	24	48.74	46.59	45.20	6.96*
49.34	47.41	46.12	**4.36**	25	49.61	47.18	45.91	7.60*

LOD ≥ 4.2 are significant and are highlighted in bold. The true top markers for weeks marked with an asterisk “*” are JAX00063853 (76.873.588 bp, LOD = 6.89) for the QTL on Chr 15 at week 12, and UNCHS041714 (11.120.784 bp) for the QTL on Chr 16 for week 24 (LOD = 7.10) and week 25 (LOD = 8.17).

**Figure 1:**
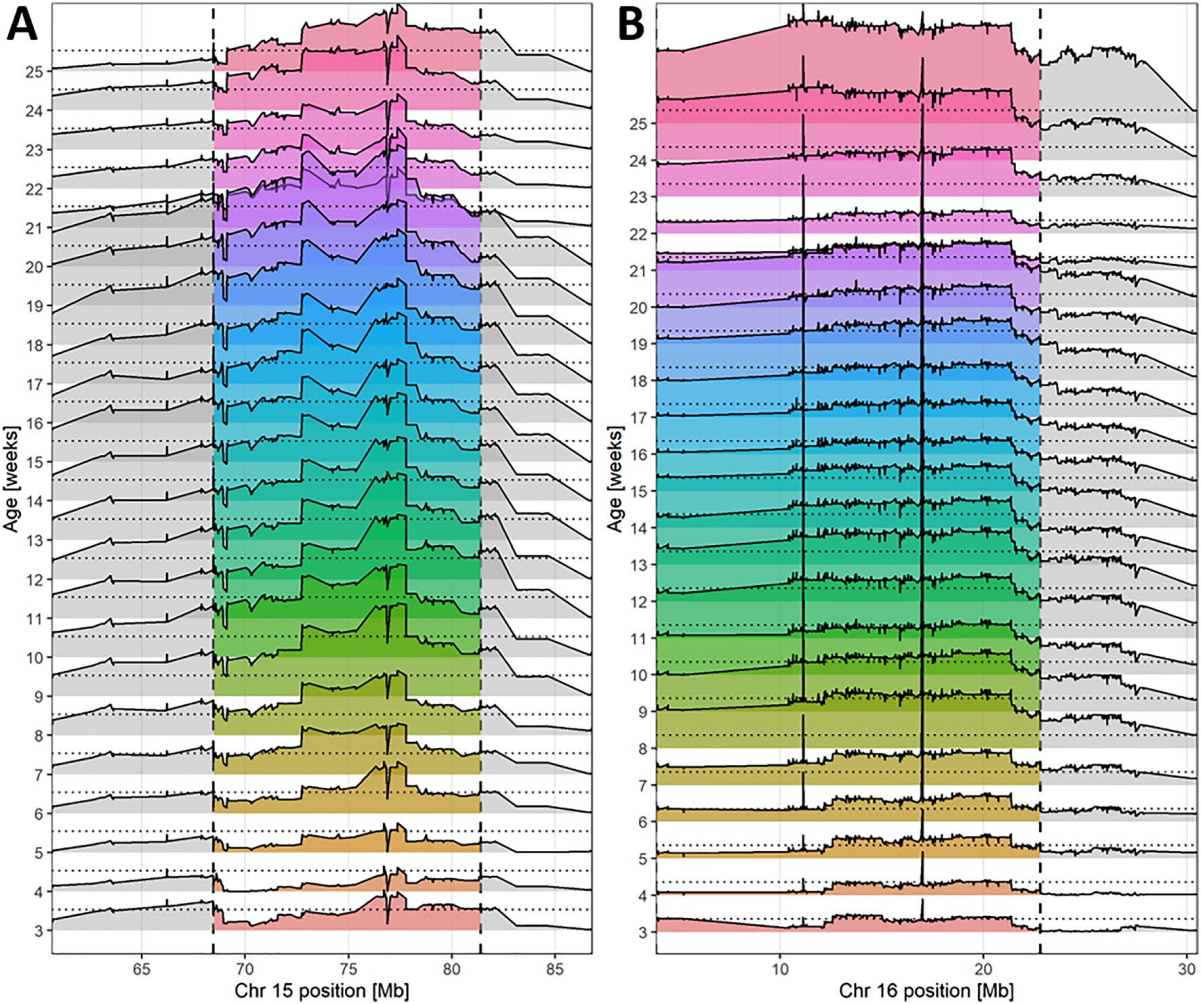
LOD curves for body weight QTLs from week 3 to 25 on Chr 15 and Chr 16. Genome-wide significant QTL regions are highlighted in color and marked by dashed vertical lines. (**A**) The QTL on Chr 15 ranged between 68.461.862 and 81.398.815 bp and (**B**) the QTL on Chr 16 ranged between 3.892.297 and 22.791.365 bp. At the markers UNC25805470 (15:68,461,862), JAX00063853 (15:76,873,588), UNCHS041714 (16:11,120,784), and UNCHS041907 (16:16,995,303), additional animals genotyped by KASP assay were available, so that the LOD curve shows sudden drops (on Chr 15) or peaks (on Chr 16).

**Figure 2:**
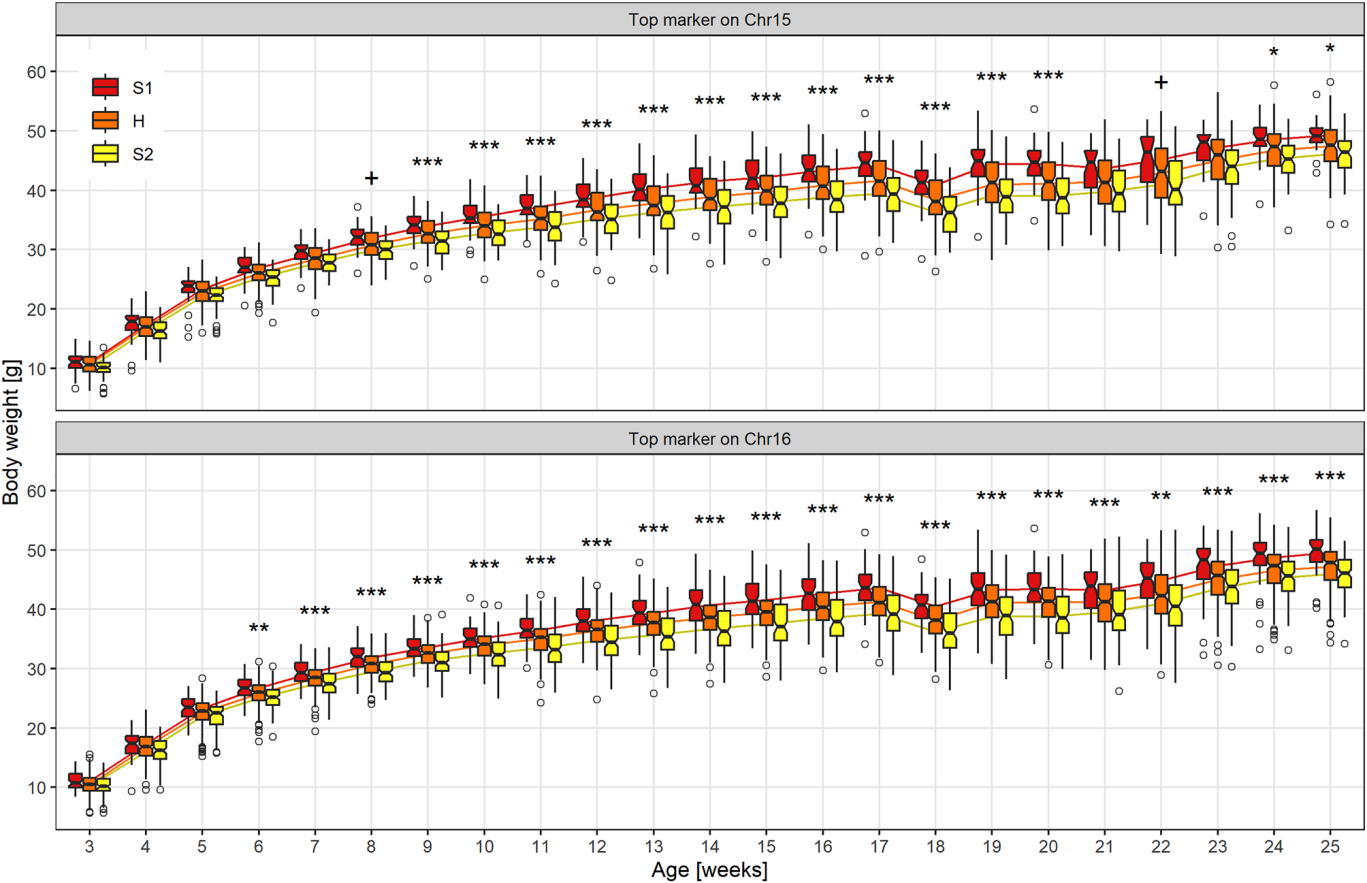
Boxplots for 397 mice of the AIL (BFMI861-S1 x BFMI861-S2) in generation 10 aged 3 to 25 weeks and curves depicting body weight development. For every time point, boxplots for all three genotype classes (S1 homozygous; H, heterozygous; S2 homozygous) are shown for SNP UNC25922623 located at the top position on Chr 15 (top) and for SNP UNCHS041907 located at the top position on Chr 16 (bottom).

In detail, a genome-wide significant QTL for body weight from week 9 to week 20 was mapped on Chr 15 between 68.46 and 81.40 Mb (Fig. 1A). The most significant SNP of this region was UNC25922623 at week 20 (77.362.610 bp; LOD = 7.81, Fig. 2 top). At this locus, the S1 allele increased body weight (Table 1). At the top SNP, homozygous S1 mice showed 5.40 g (13.8%) higher body weight (44.41 ± 4.04 g) compared to homozygous S2 mice (39.01 ± 3.76 g) and 3.24 g (7.9%) elevated compared to heterozygous mice (41.17 ± 4.47 g). Interestingly, this QTL was significant between week 9 and 20 (standard diet), then the diet was changed at week 20 (first dietary switch: high-fat, low-carbohydrate diet) and the LOD dropped below the significance level before it rose again reaching again significance for week 24 and 25. Between week 20 and 22, homozygous S2 mice gained 1.96 g, whereas homozygous S1 mice gained only 0.75 g (heterozygous: 1.78 g). During the second dietary switch (high-fat, high-carbohydrate diet, week 23 to 25), homozygous S2 mice gained 5.15 g, whereas homozygous S1 mice gained 4.18 g (heterozygous: 4.46 g). This QTL region contains 199 protein-coding genes.

Another genome-wide significant QTL for body weight from week 6 to week 25 was mapped on Chr 16 between 3.89 and 22.79 Mb (Fig. 1B). The most significant SNP of this region was UNCHS041907 at week 18 (16,995,303; LOD = 11.84, Fig. 2 bottom). At this locus, also the S1 allele increased body weight (Table 1). Homozygous S1 mice had an 11.8% higher body weight (40.45 ± 3.15 g) compared to homozygous S2 mice (36.19 ± 3.85 g) and a 6.0% higher body weight when compared to heterozygous mice (38.15 ± 3.39 g). This region contains 213 protein-coding genes.

### Candidate genes in the QTL regions

Within the confidence interval of the significant QTL on Chr 15, 10,410 SNPs and 199 potential protein-coding candidate genes are located. Due to the close relatedness between the parental lines S1 and S2, only 165 of these genes contain polymorphic DNA variants. For further analysis, also 1000 bp up- and downstream of the genes were considered. For Chr 16, this region harbours 78 SNPs and 213 genes of which 16 contain polymorphic DNA sequence variants. Mutations in these genes were scored for their potential functional effects on gene transcripts (missense mutations including SIFT score information, mutations in splice sites, UTRs, promotor, CTCF binding sites and enhancers),expression level of the encoded protein, and contribution to KEGG pathways as described in the decision tree by Delpero et al.^11^ (Supplementary Table 2). None of the candidate genes carries a loss of function mutation. Nevertheless, different mutations influencing protein sequence or gene regulation occur.

Considering the QTL on Chr 15, *Gpt* (glutamic pyruvic transaminase, soluble, upstream of the top marker: 8.12 Mb), *Cbx6* (chromobox 6, upstream of the top marker: 11.25 Mb), *Apol6* (apolipoprotein L 6, upstream of the top marker: 8.47 Mb), and *Apol8* (apolipoprotein L 8, upstream of the top marker: 9.18 Mb) ranked as top candidates (Table 2). *Gpt* was lower expressed in S1 versus S2 mice in both gonadal adipose tissue (*p* = 1.23 × 10^−7^) and liver (*p* = 2.79 × 10^−6^). Furthermore, in S1, *Gpt* possesses a deleterious missense variant in a functional domain plus several variants in UTRs, CTCF binding and splice sites, and promoter. The deleterious missense variant is caused by an amino acid exchange from isoleucine to methionine located at amino acid position 418 out of a total length of 496 amino acids. *Cbx6,* which is the second top candidate in the region on Chr 15, harbors a deleterious missense variant in a functional domain plus several variants in UTRs, CTCF binding and splice sites, and promoter in S1 mice. The deleterious missense variant results in an amino acid exchange from tyrosine to cysteine located at amino acid position 124 out of 127 amino acids (transcript ENSMUST00000148358). No expression data for this gene was available using the Clariom S assay for mouse. The apolipoproteins *Apol6* and *Apol8* were ranked third. *Apol6* was lower expressed (*p* = 3.87 × 10^−6^) in gonadal adipose tissue of S1 mice. In S1, *Apol6* carries a deleterious missense variant in a functional domain plus several variants in UTRs, enhancer and splice site. The deleterious missense variant results in an amino acid exchange from glutamic acid to aspartic acid located at amino acid position 122 out of a total length of 329 amino acids. Furthermore, *Apol6* carries a splice donor variant (rs239965506, 15:77045317_T/G) that is classified with high impact. *Apol8* was not differentially expressed in adipose tissue or liver. Besides a deleterious missense variant in a functional domain, *Apol8* in S1 mice harbours several variants in the UTRs, promotor, CTCF binding site and splice sites. The deleterious missense variant causes an amino acid substitution, changing aspartic acid to glycine acid at position 61 out of a total length of 78 amino acids (transcript ENSMUST00000229445). Five other genes in S1 mice were found to carry deleterious missense variants in the QTL on Chr 15: *Recql4* (RecQ protein-like 4, substitution of alanine to valine at protein position 967 of 1216, upstream of the top marker: 8.13 Mb), *Adgrb1* (adhesion G protein-coupled receptor B1, substitution of arginine to histidine at position 379 out of 1582, upstream of the top marker: 5.93 Mb), in *Fam135b* (family with sequence similarity 135 member B, threonine to proline at position 487 of 1403, upstream of the top marker: 2.84 Mb), *Fam227a* (family with sequence similarity 227 member A, substitution of leucine to proline at position 80 of 115 (transcript ENSMUST00000191401), upstream of the top marker: 11.03 Mb), and *Apol9a* (apolipoprotein L 9a, substitution of valine to methionine at position 151 of 310, upstream of the top marker: 8.83 Mb). Due to the diet-responsive nature of the QTL on Chr 15, genes were further scanned for ChoRE motifs, which can induce gene expression in the presence of glucose^20^. Only one gene, *Sun2* (Sad1 and UNC84 domain containing 2), carries a ChoRE-b motif (79,742,515–79,742,531 bp, motif on reverse strand CACACTCGGCCACGCG). Depending on the respective transcript of *Sun2*, this motif is either in the 5’UTR (transcripts *Sun2-201* and *-202*), 10–60 bp upstream (transcripts *Sun2-203*, *-205*, and *-208*) or more than 150 bp upstream (all other transcripts). In S1 mice, *Sun2* harbors a tolerated SNP in a functional domain accompanied by several variants in the promotor, CTCF binding site and enhancer.

**Table 2:** Top candidate genes after applying the prioritization criteria.

	Candidate gene	Chr15				Chr16		
		*Gpt*	*Cbx6*	*Apol6*	*Apol8*	*Trap1*	*Rrn3*	*Mapk1*
Type of mutation	Deleterious domain missense	✓	✓	✓	✓			
	Tolerated domain missense					✓	✓	
	UTR	✓	✓	✓	✓			✓
	Promotor	✓	✓		✓			
	Enhancer			✓				
	CTCF binding	✓	✓		✓			
	Splice site	✓	✓	✓	✓			
Gene expression	FC gonadal adipose tissue (S1/S2)	0.810	Not det	0.847	0.990	0.959	0.943	1.014
	P-value gonadal adipose tissue	**1.2E−07**		**3.9E−06**	0.531	**5.5E−05**	**2.0E−06**	**4.7E−02**
	FC liver (S1/S2)	0.965	Not det	0.989	1.002	0.995	0.917	0.999
	P-value liver	**2.8E−06**		0.503	0.887	0.426	**1.3E−06**	0.924
	Gene score	17	15	14	14	7	7	4

Bold indicates significant differences. The *p*-values are corrected according to Benjamini-Hochberg.

*FC* fold change, *Not det.* not determined.

For the QTL on Chr 16, *Trap1* (TNF receptor-associated protein 1, downstream of the top marker: 7.23 Mb), *Rrn3 (*RRN3 homolog, RNA polymerase I transcription factor, upstream of the top marker: 2.48 Mb) and *Mapk1* (mitogen-activated protein kinase 1, upstream of the top marker: 5.68 Mb) ranked highest (Table 2). The top candidate genes *Trap1* (*p* = 5.50 × 10^–5^) and *Rrn3* (*p* = 2.05 × 10^–6^) were lower expressed in gonadal adipose tissue, and *Rrn3* was additionally significantly lower expressed in the liver (*p* = 1.28 × 10^–6^) of S1 mice compared to S2. In contrast, *Mapk1* had significantly higher expression in gonadal adipose tissue of S1 mice (*p* = 4.74 × 10^–2^). S1 mice carry a tolerated missense variant in a functional domain of *Trap1* and *Rrn3*. All three top candidates carry numerous SNPs in regulatory regions potentially contributing to expression differences.

## Discussion and conclusion

To gain a deeper understanding of the differences in body weight in the two sublines of the Berlin Fat Mouse BFMI861-S1 and BFMI861-S2 that show 96.4% of genetic similarity^11^, we investigated an advanced intercross population of the initial cross between the BFMI861 mouse lines S1 and S2. Besides being genetically closely related, these two BFMI lines share the known juvenile obesity locus on Chr 3 which explains 40% of the overall variance in obesity in all BFMI lines^6^.

Performing QTL mapping on time series body weight data, we identified a QTL for body weight on Chr 15 which accounts for 9.2% of the variance in the AIL population in week 20 and another QTL for body weight on Chr 16 which explains 11.9% of the variance in week 18.

The Chr 15 locus influencing body weight has not been previously detected in BFMI mice. This QTL is genome-wide significant between week 9 and 20, is not significant afterwards and rises again during the last two weeks of the experiment. Remarkably, the drop in LOD in weeks 21, 22, and 23 coincides with a change in the diet of the mice in week 20. Until week 20, the mice received a standard diet. During week 21 and 22 the mice were fed a diet with high-fat, but very low carbohydrate content, followed subsequently by a diet high in fat and carbohydrate content for the final three weeks of the experiment from week 23 on. Under standard diet homozygous S1 gain more weight that homozygous S2. However, on the high-fat, low-carbohydrate diet this effect is opposite. The final rise in the LOD indicates that the homozygous S1 animals catch up with the homozygous S2 animals in body weight gain again and reestablish the initial difference between the two genotypes. This LOD drop and rise could indicate different responses to the diet change depending on the genotype especially during the first diet switch. The gene *Sun2* on Chr 15 is an interesting candidate to implement this diet responsiveness via its ChoRE motif. Albeit no SNPs between S1 and S2 were located directly in the ChoRE motif, the gene encompasses many SNPs in the promotor, enhancer or CTCF region potentially leading to an altered gene regulation or transcript variant mediating difference in diet responsiveness. Female mice with a homozygous *Sun2* knockout show a significant decrease in lean body mass (https://www.mousephenotype.org). It can be speculated that the homozygous S1 mice are less flexible in substrate metabolization and probably need both, fat and carbohydrates, to increase body weight further. This is in line with the fact that inbred S1 mice are lipodystrophic with lower adipose tissue weight and elevated liver weight and liver fat content^10^. Therefore, this QTL likely contributes to the metabolic difference between the parental lines S1 and S2. The analysis of time-series body weight data allowed the mapping of QTLs which are acting during specific time periods only and that could be hidden at later age or under specific conditions such as dietary changes. Such developmental stage-dependent gene activity could play an important role in adult body weight variation.

The QTL on Chr 16 had been mapped previously for body weight at the endpoint of week 25 exactly at the same position in the same AIL population BFMI861-S1 × BFMI861-S2^11^. In the current study, we associated this QTL also with body weight at younger age from week 6 until week 25. The highest LOD score in this study was 11.8 in week 18 compared to the previous study with 7.1 at 25 weeks, where the same AIL population was used^11^. This indicates as postulated and shown in other studies^21^ that the effect varies over time and that QTLs can fade or remain undetected if single time points are investigated.

The top candidate genes for the novel QTL on Chr 15 associated with body weight are *Gpt*, *Cbx6, Apol6* and *Apol8*.

*Gpt* encodes for alanine aminotransferase 1 (ALT1) and plays a crucial role in the transamination of amino acids, channeling them into gluconeogenesis and the urea cycle. A deleterious missense variant in a functional domain could result in impaired ALT1 function in S1 mice, potentially disrupting amino acid metabolism in various tissues, such as the liver and adipose tissue which, in turn, might have repercussions forgrowth and body weight. *Cbx6* (chromobox 6) is predicted to be involved in the regulation of transcription. Knockout mice of *Cbx6* have an increased lean mass and decreased blood glucose level (https://www.mousephenotype.org) pointing towards an involvement in growth and metabolism. A deleterious missense variant in a domain of *Cbx6* in S1 mice could increase body weight of S1 mice. The gene *Apol6* encodes for the lipid binding protein APOL6 (apolipoprotein L6) which acts extracellularly as part of high-density lipoproteins and intracellularly affecting lipid transport and binding to organelles^22^. Overexpression of *Apol6* results in induced mitochondria-mediated apoptosis^23^. Further, APOL6 is described to be involved in the regulation of the differentiation of 3T3-L1 adipocytes^24^. Thereby, dysfunctional APOL6 and a reduced expression in adipose tissue in S1 mice could contribute to a malfunctional adipose tissue of these mice resulting in the metabolic unhealthy phenotype with elevated liver weight of S1 mice. *Apol8* is a metabolically less studied member of the apolipoprotein family, which was shown be involved in neuronal differentiation^25^ and differentially expressed in stretched myocytes^26^.

For the QTL on Chr 16, *Trap1* (TNF Receptor Associated Protein 1) and *Rrn3* (RNA polymerase I transcription factor homolog) were ranked as top two candidate genes which have been previously described in Delpero et al.^11^ to be associated with final body weight at week 25. The protein TRAP1 is localized to the mitochondria and regulates metabolic reprogramming and mitochondrial apoptosis^27^. Furthermore, *Trap1*-knockout mice show reduced body weight^28^ indicating that altered *Trap1* regulation could be involved in metabolic changes in S1 mice and thereby alter body weight development. *Rrn3* is highly conserved between yeast and mammals^29^. In yeast, RRN3 is required for the transcription of rRNA by RNA polymerase 1^30^ and in mammals its phosphorylation regulates ribosome biogenesis^31^. Thereby, sequence variation of *Rrn3* could affect protein synthesis and result in body weight differences. Furthermore, *Mapk1* (mitogen-activated protein kinase 1) is ranked subsequently. MAPK1 is an extracellular signal-regulated kinase (ERK) and acts in a wide variety of cellular processes including proliferation, differentiation and transcription. MAPK1 is regulated by phosphorylation; different splice isoforms exist^32^ and sequence variation, e.g. in the 5’UTR, may result in differently spliced transcript variants. Consequently, sequence variation has the potential to modify protein function, potentially leading to changes in body weight.

While the juvenile obesity QTL on Chr 3 is responsible for juvenile obesity until about 8 weeks in all BFMI lines^6^, the QTL on Chr 16 in our current study affects the persistence of obesity in the BFMI861-S1 mouse line from week 6 until week 25. Further studies are needed to clarify whether and how these genes and their regulation could influence body weight gain.

In the current study, we could identify one novel QTL and one previously identified QTL for body weight in our population by using time series data. The identification of these two QTLs which are significant over a wide range of ontogenetic development help us to unravel the genetic puzzle that is driving the higher body weight observed in the BFMI lines over time.

Body weight is influenced by many factors including environmental as well as genetic factors. While some genes are known for their significant influence on body weight when they dysfunction (e.g. leptin, leptin receptor, *MC4R*, *POMC*), most genes have a small impact and many more are still undiscovered^2^. Genes with smaller effects are harder to track, in particular when their effect is time-dependent so that they mainly act during certain time periods such as puberty. Obesity is a complex trait driven by multiple genetic and environmental factors. While many environmental factors are well known, the contribution of many genetic factors and the interaction between genetic determinants and environment is currently highly investigated in the field of nutrigenetics^33^. Obesity is a disease that develops over time, where puberty and young adulthood are very sensitive phases for disease onset and progression^34^. QTL mapping under different conditions such as time series data, genetic background and dietary condition and the subsequent identification of genomic regions and candidate genes influencing obesity in both mice and humans are important to help understanding the genetic contribution and its interaction with environmental factors to this common complex human disease.

### Supplementary Information

Supplementary Table S1.

Supplementary Table S2.

Supplementary Information 1.

Supplementary Information 2.

## Data Availability

DNA sequencing data were deposited at the NCBI Sequence Read Archive (SRA) under BioProject ID: PRJNA717237 and is available at: https://www.ncbi.nlm.nih.gov/bioproject/717237. BFMI861-S1 gene expression data measured with Clariom™ S Assay for mouse were deposited at the ArrayExpress Archive (accession E-MTAB-11512).
